# The proliferation of medical schools in Brazil: a threat to the quality of medical education?

**DOI:** 10.1016/j.bjorl.2023.101354

**Published:** 2023-10-27

**Authors:** José Eduardo Lutaif Dolci

**Affiliations:** School of Medical Sciences, Santa Casa de São Paulo, Brazil

As everyone knows, in 1910, Abraham Flexner published the famous Flexner Report on medical schools in the USA, which promotes a great revolution in the study of medicine in American medical schools and, why not say, the world.^1^

We currently have 389 medical schools in Brazil, second only to India with 392, but with 1.4 billion inhabitants and Brazil 214 million.^2^

The indiscriminate opening of medical schools in our country aims to supply the false premise that there is a lack of doctors in the interior of Brazil. With more than 570,000 doctors, Brazil already has 2.6 professionals per 1,000 inhabitants, surpassing countries such as Japan, the USA, Canada, Chile, Mexico, and other first-world countries. It is more than proven that the lack of doctors in smaller cities and far from large centers is a reflection of the absence of public policies capable of keeping these professionals in these locations (here we understand the lack of a State Career Progression Program for Doctors), lack of good working conditions, decent wages and security for themselves and their families. There are available doctors to work in the most distant corners; what is lacking is the political will to establish conditions for this to happen. Since 2014, with the new National Curriculum Guidelines for the Medicine courses of the "More Doctors program," several attempts to place doctors in Brazil's small and distant cities have proven ineffective, with very large financial and scientific losses.^3^

By 2035, our country will have more than 1 million active doctors, which gives a scenario of 4.43 doctors per 1000 inhabitants. However, if exceptional measures are not adopted (here we understand again the creation of the State Career Progression Program for Doctors), the inequality of geographical distribution will be maintained or even aggravated, which will make the localized shortage of professionals persistently even in a scenario of greater and growing supply of doctors. It is important to remember that the indiscriminate opening of medical schools in Brazil can negatively affect the quality of medical education.^4^

Apart from this, the lack of qualified professors and preceptors for medical education is directly related to the lack of public policies to retain these professionals in universities and university hospitals. The training of professionals without the minimum qualification required for the exercise of the profession, based on efficiency, quality, and safety, will generate an overload of problems, not to mention errors in diagnosis and conduct that, in the short term, will be evidenced in the costs of our already weakened SUS (Unified Health System, public Brazilian Health Insurance program).

We need to create ways to evaluate students and medical schools. The systematization of periodic evaluations (serial tests or progress tests) and end-of-course evaluation (along the lines of REVALIDA) are urgently debated alternatives. Again, a minimum qualification should be required.

According to the study "Medical Demography in Brazil," recently released by the Brazilian Medical Association (AMB), between 2010 and 2023, more than 250 thousand doctors entered the job market, and in 2000, the country had 239 thousand doctors. This shows that while the number of professionals has more than doubled, the general population has grown by around 27%.^4^

This scenario indicates that many medical schools are heading towards an implacable reality: the decline in the quality of medical school graduates.

There are more and more problems in these new medical schools, but two are fundamental to point out:

1 Lack of internship (training) fields since the training of medical students takes place mostly outside the classrooms, especially in internship fields. No simulation is a substitute for actual patient contact in presence. Real supervised attendances are a unique opportunity to evaluate the development of students critical thinking and whether they are fit for professional practice. Internships should take place at all levels of complexity, as determined by the national medical curriculum guidelines.

Internships in hospitals are indispensable and a minimum of effectively occupied beds must be guaranteed and monitored so that there is no overlapping of courses in the same beds. UBS, basic public outpatient clinics, UPAS, secondary public outpatient clinics, and hospitals must be eligible to receive students, including accreditation as a teaching hospital.

The SUS continues to be the ideal field of practice, so it needs to be adapted to receive students, professors, and preceptors.

2 Lack of qualified professors and preceptors for medical education. This lack is directly related to the lack of policy and financial incentive for qualified teachers for such a role. The training of professionals without the minimum qualification required for the exercise of the profession, based on efficiency, quality, and safety, will generate an overload of problems, not to mention errors in diagnosis and conduct that in the short term will be evidenced in the costs of our already weakened SUS (public Unified Health System)

We need to create programs for evaluation medical students and medical schools. The systematization of periodic evaluations (serial tests or progress test) and end-of-course evaluation (along the lines of REVALIDA) are alternatives to be urgently debated. Reflecting on the great financial investment that is made in the young egress is a pertinent discussion, but much more important is the reflection on the quality of care for the Brazilian population, so a minimum qualification should be required.

Finally, the proliferation of medical schools without minimum conditions to train medical professionals in an increasingly competitive labor market reflects the great financial interest related to the high cost of training young doctors to the detriment of the population.

## Conflicts of interest

The authors declare no conflicts of interest.
